# Work Environment and Work Context Factors Associated With Homecare Workers' Intention to Leave: An Analysis of a National, Multicenter Cross-Sectional Study

**DOI:** 10.1155/jonm/1554741

**Published:** 2025-07-09

**Authors:** Nathalie Möckli, Barbara Villiger, Michael Simon, Carla Meyer-Massetti, Franziska Zúñiga

**Affiliations:** ^1^Institute of Nursing Science, Department of Public Health, University of Basel, Bernoullistrasse 28 CH-4056, Basel, Switzerland; ^2^Clinical Pharmacology & Toxicology, Department of General Internal Medicine, Inselspital – University Hospital of Bern, Freiburgstrasse CH-3010, Bern, Switzerland; ^3^Institute for Primary Health Care BIHAM, Faculty of Medicine, University of Bern, Mittelstrasse 30 CH-3012, Bern, Switzerland

**Keywords:** “Health Workforce”[Mesh], Home care services [Mesh], “Home health Nursing”[Mesh], “long-term Care”[Mesh], “personnel Turnover”[Mesh], working conditions [Mesh]

## Abstract

**Background:** Given the projected shortage of homecare workers, it is important to examine and optimize working conditions in order to keep them in their field of expertise. However, there is limited knowledge about the prevalence of the intention to leave their job and the modifiable factors that contribute to it.

**Aims:** We assessed the prevalence of homecare workers' intention to leave their current job or the homecare sector entirely and the relationship between work environment, i.e., organizational characteristics (e.g., leadership, staffing, teamwork, and predictability) and work context factors, i.e., job characteristics and role definition (e.g., autonomy, overtime, and role clarity) with leaving intentions.

**Methods:** This analysis included data from a national, multicenter, cross-sectional study in Switzerland, conducted from January to September 2021. We included 1898 homecare workers of 85 stratified randomly selected homecare agencies, performing descriptive analyses and logistic multilevel regressions to calculate prevalence and examine associations.

**Results:** Overall, 58.5% of respondents reported at least a slight intention to leave the current job, while 12.6% considered leaving the homecare sector. Leadership was significantly negatively associated to respondents' intentions to leave the current job; predictability and job satisfaction were significantly negatively associated with both leaving intentions. Job satisfaction mediated work environment factors such as staffing.

**Conclusions:** Leadership was one of the most important work environment factors related to intention to leave. Therefore, political decision makers and homecare managers need to lay the foundation to foster development, improvement, and empowerment of leadership roles. Homecare agencies should target improvement efforts based on the effect of overtime and the implementation of predictability and social support in their organization in order to reduce intention to leave and improve job satisfaction. Future studies could focus on deepening our understanding by empowering nurse leaders in creating and sustaining a positive work environment.

## 1. Introduction

Demand for homecare is increasing due to an aging population, increasing chronic health conditions, shorter hospital stays, and the desire of people with care needs to remain at home as long as possible [1–3]. As a result, the need for homecare workers is growing and expected to further grow in the upcoming years, as seen by numbers from the United States, where the workforce needs to grow by 34% from 2019 to 2029 [4] and in Switzerland by 57% from 2014 to 2030 to cover expected demands [5]. Given this development, a shortage of homecare workers is predicted [1, 5, 6]. This shortage is further complicated by the limited number of young nurses entering the homecare sector [1], the high number of nurses retiring, and the circumstance that many nurses leave the profession prematurely [6, 7]. International data show that especially the early withdrawal from the profession and a high intention to leave among homecare workers represents a major challenge. In Canada, e.g., one in 10 and in the United States and Japan one in four homecare workers are thinking about leaving their job [8–11].

Future demand for homecare services can only be met if sufficient homecare workers are available. Therefore, it is critical to address not only how to increase the number of nurses entering the homecare workforce but also how to retain those already in the homecare workforce. Hence, it is important to identify modifiable factors that are associated with the intention to leave the homecare job or the profession. However, we know little about such factors in the homecare sector; a setting that is very different from institutional care.

Homecare in general refers to formal care services provided by professional homecare workers in the clients' own homes [12]. Homecare workers are responsible for, but not limited to, assessing needs and plan care, assisting clients with activities of daily living (e.g., body care), supporting family caregivers, collaborate with other healthcare professionals, and taking preventive, technical, and diagnostic medical measures [13]. In contrast to institutional care, e.g., nursing homes, homecare is provided on a periodic basis in the clients' home, where homecare workers are guests and need to adapt care delivery to the environment [12, 14]. They also mostly work alone and have limited direct contact with colleagues and other healthcare professionals involved in clients' care [15]. Given these differences, homecare workers' intention to leave may be influenced by different work-related factors than those of care workers in institutional settings such as hospitals or residential long-term care.

The concept of intention to leave originates in the theory of reasoned action by Fishbein and Ajzen [16], which describes the relationship between the intention and the behavior: the stronger the intention for something (e.g., intention to leave), the higher the likelihood of this behavior (e.g., staff turnover). So far, several researchers have confirmed that intention to leave is a precursor of staff turnover [17–20], with turnover generally understood as the termination of employment in terms of leaving an organization or moving to a different unit within an organization [21].

**Figure 1:**
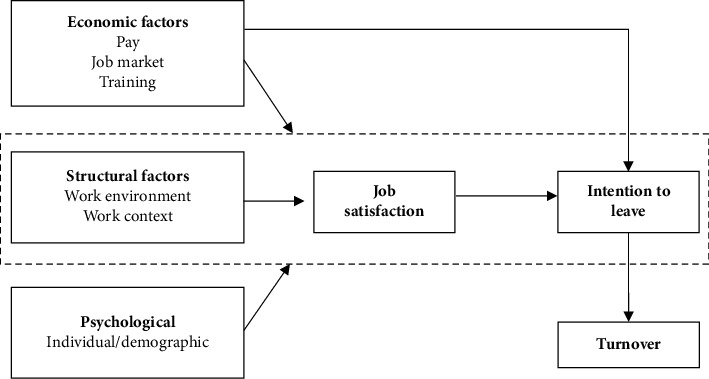
Model of nurse turnover behavior. *Note*. The model derives from “Job satisfaction and turnover among nurses: integrating research findings across studies” by Irvine and Evans, 1995, Nursing Research, 44(4), p. 247. The dashed rectangle marks the study area for this analysis.

For this analysis, we used the model of nurse turnover behavior (c.f.  Figure 1), which was developed by Irvine and Evans [22] in the hospital setting, as there is no model for the homecare sector available. The model distinguishes economic, structural, and psychological factors that influence nurse turnover, assuming that these factors are related to job satisfaction, which mediates the effects on nurses' intention to leave. Based on the concept, they concluded that hospital nurses with leaving intention were most likely to turnover.

In this analysis, we focused on structural factors, that is, work environment and work context factors (dashed rectangle in Figure 1), as Irvine and Evans [22] concluded that these factors had a higher association with job satisfaction and intention to leave than economic or psychological factors. Additionally, homecare agencies have more control over structural factors and can directly modify factors that are associated with homecare workers' intention to leave. Irvine and Evans [22] did not define the work environment; therefore, theye use the definition of Lake [23], who defines the work environment “as the organizational characteristics of a work setting that facilitate or constrain professional nursing practice” (p. 178) (e.g., leadership and staffing). Work context in turn is described by Irvine and Evans [22] as job content in terms of job characteristics (e.g., routinization and autonomy) and role definition (e.g., defined responsibilities). According to Irvine and Evans [22], job satisfaction is strongly negatively associated with intention to leave, which has been confirmed in different homecare studies [9, 24, 25].

So far, the literature shows that work environment factors such as work overload [26], work-related injuries [10, 25, 27], lack of career advancement opportunities [28, 29], lack of respect from the agency or supervisor [30], or team conflicts [8, 24, 29, 31] were associated with higher intention to leave the job among homecare workers. In contrary, homecare workers' intention to leave the job was lower when the level of agency support [8, 10, 32] and quality of leadership was higher [8, 26, 28, 33, 34] as well as when adequate staffing [32] and clear structures (e.g., clear instructions and meaningful staff scheduling) [28] were present.

Although predictability (e.g., prior information about changes and receiving all information to perform the job well) is crucial in organizing, planning, and delivering care to clients in their homes, it has not yet been studied in relation to homecare workers' intention to leave. Similarly, a perceived safe work environment (e.g., reduced risk of patient harm due to organizational efforts) was positively associated with hospital nurses' intention to stay in their current job [35, 36] but has not been explored so far in the homecare setting.

In terms of work context factors, a positive work and family-oriented culture, such as employers' understanding of the employees' family roles [14] was associated with lower homecare workers' intention to leave the organization and the profession. Similarly, more autonomy, such as participation in decision-making or flexibility in task organization, was negatively associated with intention to leave the job [29], the profession [37], or even the healthcare sector [38].

To our knowledge, the association between homecare workers' intention to leave and overtime, role conflicts, or role clarity has not yet been studied. However, in other healthcare settings, overtime (i.e., working more than contracted hours) was associated with lower care workers' intention to stay [39]. Also, role conflicts (i.e., conflicting demands or expectations) [15] were positively associated with work stress and turnover among hospital nurses [40].

Given the increasing demand for homecare workers, a comprehensive understanding of modifiable factors associated with homecare workers' intentions to leave is needed. Work environment factors such as predictability, safety climate, and work context factors such as overtime, role clarity, and role conflicts have not yet been studied in relation to homecare workers' intention to leave, although they represent central and adaptable factors in homecare.

### 1.1. Aims

We therefore aimed at the following:

1. To assess the prevalence of homecare workers' intention to leave the current job and their intention to leave the homecare sector entirely
2. To explore the association of work environment factors (i.e., leadership, staffing, teamwork, social support from colleagues, predictability, and safety climate) and work context factors (i.e., role clarity, role conflicts, and overtime) with homecare workers' intention to leave the current job and the homecare sector entirely

## 2. Materials and Methods

### 2.1. Study Design

This study is a substudy of the SPOT^nat^ study (homecare coordination and quality—a national study), a national, multicenter, cross-sectional Swiss homecare study [41].

### 2.2. Setting and Sample

For the SPOT^nat^ study, a random sample of 88 of the approximately 931 public and private Swiss homecare agencies [42] was used, stratified according to seven geographical regions of Switzerland. Agencies with less than 10 employees were excluded. Within each homecare agency, all homecare workers who met the following criteria were invited to participate: (1) employed for at least 3 months in the participating agency, (2) minimum of 18 years of age, and (3) able to read and understand German, French, or Italian. For large agencies (≥ 100 employees), a randomized sample of 100 employees was offered to reduce study burden. Detailed information about the setting, sampling, and sample size calculation can be found in the SPOT^nat^ study protocol [41].

For this analysis, we included only homecare workers involved in direct client care (i.e., conducting needs' assessments and providing basic client care and/or treatment) with one of the following educational backgrounds: (a) graduate nurses with 3 to 4 years of tertiary level education, qualified for coaching and needs assessments, that is, registered nurses (RNs) including those with a masters' degree; (b) basic nurses with a 3-year vocational training, qualified for additional competencies, for example, licensed practical nurses (LPNs); (c) nursing assistants with Swiss Red Cross course certificate, without additional competencies, for example, homecare aides (HCAs). Students, trainees, and homecare workers with a leadership function (e.g., head nurse) were excluded.

### 2.3. Variables and Measurements

#### 2.3.1. Dependent Variables

We assessed intention to leave the current job with three items, as recommended by Zúñiga et al. [43] and used by Gaudenz et al. [44]. Two items “I often think about quitting” and “I will probably look for a new job next year” originate from the turnover subscale of the Michigan Organizational Assessment Questionnaire (MOAQ) [45], the third item “I am currently looking for another job” originate from Mobley et al.'s study [46]. Items were rated on a 5-point Likert scale ranging from “0 = *strongly disagree*” to “4 = *strongly agree*” and showed good internal validity in the nursing home study, with Cronbach's *α* = 0.91 [44]. We calculated the sum over all items (ranging from 0–12) according to Gaudenz et al. [44], where higher scores indicated higher leaving intention; respondents with missing values in one or more items were removed to build the sum score (*n* = 54).

Intention to leave the homecare sector entirely was assessed with an investigator-developed single-item “I often think about giving up my work in homecare entirely” (translated from German). Answer options ranged from “0 = *strongly disagree*” to “4 = *strongly agree*” on a 5-point Likert scale, with higher scores indicating higher intention to leave the homecare sector entirely.

#### 2.3.2. Independent and Mediator Variables

For work environment factors, we assessed leadership, staffing and resource adequacy [23], teamwork, safety climate [47, 48], predictability, and social support from colleagues [49, 50]. For work context factors, we assessed role clarity, role conflicts [49, 50], and overtime [51]. We assessed job satisfaction with the Copenhagen Psychosocial Questionnaire II (COPSOQ II) [50, 52]. Detailed information on these variables can be found in Table 1. Where available, we used validated translations of the scales for the French [54, 55] and Italian [55–57] versions of the questionnaires. For some subscales, the internal consistency of the German-language versions was tested in the Swiss homecare setting [53] and is shown accordingly in Table 1.

#### 2.3.3. Control Variables

We assessed individual and agency factors. For individual factors, we included educational background (RNs, LPNs, and HCAs), as well as age (years), employment percentages (number), and compensation (hourly wage or annual salary) because in previous studies, older homecare workers [8, 26, 27, 31] and those with full-time jobs or annual salaries [11] were significantly less likely to leave. For agency factors, we included agency size (number of full-time equivalents and number of clients per year), catchment area (rural, suburban, or urban), and profit status (public nonprofit, private nonprofit, and private for-profit) because in previous studies, working for a for-profit agency was significantly positively associated with the intention to leave [8, 27].

### 2.4. Ethical Consideration and Data Collection

Following the declaration of no objection (Req-2020-00110) from the lead ethics committee of Northwestern and Central Switzerland (EKNZ), data collection for the SPOT^nat^ study took place between January and September 2021. Each homecare agency signed an informed consent for study participation. The agency questionnaire was completed by the management (either as paper or electronic version). The paper–pencil employee questionnaires were sent to the participating agencies for internal distribution and included a prestamped return envelope addressed to the research team to ensure the confidentiality of the completed questionnaires. The first page of the employee questionnaire contained information about the purpose of the study, highlighting the completely voluntary and anonymous participation, and informed consent by returning the completed questionnaire. Each agency was given 9 weeks to complete the data collection. We informed the agencies about the response rate after 3 and 6 weeks, respectively, and at the end of the data collection. Each time we offered material and discussed measures with the management to increase the response rate, such as information and reminder leaflets, presentation of the study, and objectives for team meetings or allowing the completion of questionnaires to count as working time.

### 2.5. Data Analysis

For descriptive statistics and Aim 1, we calculated numbers, percentages, means, standard deviations, and ranges. Scales of independent and mediator variables were calculated using the valid mean substitution [58]. We checked for inter item consistency (i.e., Cronbach's alpha), missing data, normal distribution, outliers, floor, and ceiling effects.

Due to the highly left-skewed distribution of intention to leave the current job, we prepared the variable in two ways. According to Gaudenz et al. [44], the sum score variable (range 0–12) was dichotomized with a cutoff set at 1 to 0 = “*fully disagreed intention*” and 1–12 = “*agreed with at least a slight intention to leave the current job.*” For the sensitivity analysis in Aim 2, however, we also ran a multilevel linear regression model, for which we built a mean score of the three items with values ranging from 0–4 and higher scores meaning higher intention to leave the current job.

Since intention to leave the homecare sector entirely (i.e., single-item) also showed a left-skewed distribution, we dichotomized it according to its response options into 0–2 = “*disagree with intention*” and 3–4 = “*agree with intention to leave the homecare sector entirely.*” For the sensitivity analysis in Aim 2, we also conducted a multilevel linear regression model here, leaving the response options of the single item in its initial form.

**Figure 2:**
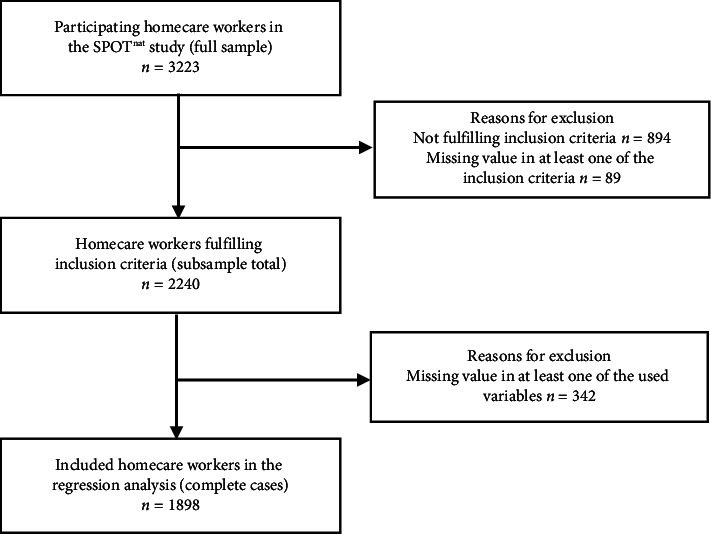
Flowchart of the number of participants included and lost in each step. Note: See Table 2 and Appendix A for the missing values. Homecare workers involved in direct client care were included in the subsample. Students, trainees, and homecare workers with a leadership function (e.g., head nurse) were excluded.

For Aim 2, we created a complete case dataset for the variables under study. Of the 3223 participating homecare workers (response rate 74%) of the 88 participating homecare agencies, a total of 2240 respondents met the inclusion criteria for this analysis (see the flowchart in Figure 2). After building a complete case dataset, i.e., removing the missing values by listwise deletion, for the variables under study, a sample of 1898 respondents in 85 homecare agencies remained for the data analysis. This resulted in a loss of 15% of respondents, although none of the variables had more than 2.9% missing values. To be as transparent as possible, we calculated the descriptive statistics for the total subsample (i.e., all respondents who met the inclusion criteria) and for the complete case dataset, which can be found in Appendix A.

To explore the relationship between structural factors and intention to leave, we calculated two logistic multilevel regression models using the R package “lme4” [59]. To assess, whether a multilevel model was indicated, we calculated the Intraclass Correlation Coefficient 1 (ICC1) [60] using the R package “rptR” [61]. The ICC1 for intention to leave the current job was 0.09 and for intention to leave the homecare sector entirely 0.03. Although ICC1 for intention to leave the homecare sector entirely was below the commonly used threshold of 0.05, we calculated a multilevel model due to the nested design [62, 63]. For initial variable selection and to examine the contribution of the independent variables to the dependent variable (i.e., intention to leave the current job/intention to leave the homecare sector), we entered each of the nine independent variables in a separate univariate multilevel regression model and screened it for criterion of *p* < 0.20 [64]. We then combined all independent variables *p* < 0.20 and all control variables in one model. As the last step, we added the mediator variable (satisfaction) to create the final model. To show the explained variance by the variables included in the models, we calculated the marginal *R*^2^ (i.e., consider the variance of the fixed effects) and conditional *R*^2^ (i.e., consider the fixed and random effects) [65]. We calculated odds ratios (OR) and 95% confidence intervals (CI). We also tested for multicollinearity by calculating the variance inflation factor (VIF) [66, 67]. The values of the VIF were all around one, except for two variables (profit-status and number of clients), where values were around 3.5 indicating no multicollinearity. Finally, we performed the sensitivity analysis as described above by running the linear multilevel regression model for intention to leave the current job and intention to leave the homecare sector. We conducted the data analysis using statistical software environment R Version 4.1.0 [68].

## 3. Results

### 3.1. Respondent Characteristics

Most respondents were RNs (45.1%), female (94.1%), and with a mean age of 46 years (SD = 11.9). Most of the respondents had more than 10 years of professional experience (59.9%) and had been working at the current agency for five or fewer years (53.9%).  Table 2 shows the descriptive statistics of the respondents included in the regression analysis.

### 3.2. Prevalence of Intention to Leave

Overall, 58.4% of the respondents agreed to at least a slight intention to leave (sum score ≥ 1) the current job and around 12.0% of respondents slightly or strongly agreed to the intention to leave the homecare sector entirely. For more details on the prevalence of intention to leave, see Table 3.

### 3.3. Characteristics of the Independent and Mediator Variables

Most of the independent variables (i.e., structural factors) had acceptable to good inter item consistencies, with Cronbach's *α* > 0.70, except for the variables' social support from colleagues (*α* = 0.61) and role conflicts (*α* = 0.69). For descriptive results on these variables, see Table 3.

### 3.4. Structural Factors Associated With Respondents' Intention to Leave the Current Job

Table 4 includes the results of the logistic multilevel models for respondents' intention to leave the current job. In the univariate multilevel models, all structural factors were significantly associated to intention to leave the current job. The best model fit showed the adjusted model including job satisfaction (AIC = 1912), whose variables also explained the largest proportion of the variance in intention to leave the current job (marginal *R*^2^ = 0.50 and conditional *R*^2^ = 0.52).

In the final model, significantly negatively associated to respondents' intention to leave the current job were the work environment factors' “leadership” (OR = 0.48, CI [0.36, 0.65], *p* < 0.001) and “social support by colleagues” (OR = 0.99, CI [0.99, 1.00], *p* > 0.05), the latter showing only a small effect (i.e., OR close to 1). In regard to the work context factor, we only found a significant association with the variable “overtime.” Having to work overtime “almost every shift” (OR = 2.24, CI [1.15, 4.38], *p* < 0.05) or “every 2–4 working days” (OR = 2.05, CI [1.11, 3.82], *p* < 0.05), compared to never, was positively associated to intention to leave the current job. The work environment factors' “staffing” and “predictability” were significantly associated to intention to leave the current job in the model without the mediator variable “job satisfaction,” while this relationship was completely mediated when “job satisfaction” was added, while “job satisfaction” (OR = 0.93, CI [0.91, 0.94], *p* < 0.001) was significant in the final model.

The sensitivity analysis showed similar results in strength and direction of the associations (see Appendix B).

### 3.5. Structural Factors Associated With Respondents' Intention to Leave Homecare

Table 5 includes the results of the multilevel models for respondents' intention to leave the homecare sector entirely. In the univariate multilevel models, all structural factors were significantly related (*p* < 0.05), except overtime (Table 5, first row). However, since overtime met the screening criterion (*p* < 0.20), all structural factors were added to the adjusted regression. The best model fit showed the adjusted model including job satisfaction (AIC = 1185), whose variables also explained the largest proportion of the variance in respondents' intention to leave the homecare sector entirely (marginal *R*^2^ = 0.31 and conditional *R*^2^ = 0.33).

In the final model, the only significant negative association to respondents' intention to leave the homecare sector entirely showed the work environment factors' “leadership” (OR = 0.72, CI [0.53, 0.99], *p* < 0.05) and “predictability” (OR = 0.99, CI [0.98, 1.00], *p* < 0.05), the latter with only a small effect. In this model too, the mediator variable “job satisfaction” (OR = 0.95, CI [0.94, 0.96], *p* < 0.001) was significantly negatively associated with the intention to leave the homecare sector entirely. The work environment factor “staffing” was significantly associated in the adjusted model without the mediator variable “job satisfaction.” However, when the mediator variable was added, the association became insignificant. None of the work context factors (i.e., role clarity, role conflicts, and overtime) were significantly related to respondents' intention to leave the homecare sector neither in the adjusted nor in the final model.

A sensitivity analysis using linear regressions (see Appendix B) showed comparable results in the strength and direction of the associations.

## 4. Discussion

The aim of this study was to explore factors related to homecare workers' intention to leave the current job or homecare sector entirely. To our knowledge, this was the first study in Switzerland to examine the prevalence and related factors of homecare workers' intention to leave the job or the homecare sector entirely and the first study to investigate if specific factors such as predictability, safety climate, role clarity, and role conflicts are related to intention to leave.

We found a high prevalence of intention to leave the current job (58.5%), which was certainly due to the high sensitivity by using the calculation of Gaudenz et al. [44] (i.e., assessing at least a slight intention to leave the current job vs. no intention at all) and was comparable to the prevalence of 56% they reported in nursing home employees in Switzerland. The prevalence of intention to leave the homecare sector entirely, which was measured with a single item, was found to be 12% in our study (note that this prevalence cannot be compared with respondents' intention to leave the current job due to different measurement methods, i.e., scale vs. single item). Other studies that measured intention to leave with a single item (and response option yes or no) reported an intention to leave the current job of 26.2% [9] and 15.6% [69] in Japan and Korea, respectively. However, these studies focused on intention to leave the current job and not the homecare sector. In regard to leaving the homecare sector, a study from Germany reported that over 1/3 of homecare and nursing home nurses thought of leaving the nursing profession [24].

Of the tested work environment and work context factors in our study, leadership showed the strongest association to intention to leave the current job and also to intention to leave the homecare sector entirely. This association has also been found in several previous studies [28, 33, 34, 70]. Senek et al. [70] reported in their very recently conducted UK homecare study that sufficient support by the management was significantly negatively associated to intention to leave. When it comes to leadership styles, the transformational style is seen as most promising, showing positive effects not only on staff outcomes but also on organizational and patient outcomes [71]. This style focuses on the employees' development, improvement, and empowerment [72]. Transformational leaders act as role models, create common visions, motivate by providing meaning and challenges to tasks, stimulate innovation and creativity, act as mentors, and provide learning opportunities in a supportive climate for growth [72]. Studies in homecare show that good leadership is characterized by recognition of good work [8] or frequent, constructive feedback [34], while the latter was even associated with increases in homecare workers' self-efficacy. Therefore, people in leadership positions in homecare agencies play a key role in reducing their employees' intention to leave by using their leadership skills not only to strengthen employees' decision-making skills but also to increase their autonomy [29, 73].

Social support by colleagues and work overtime only was significantly associated with intention to leave the current job, while predictability only with intention to leave the homecare sector as a whole. However, social support by colleagues and predictability showed only small effects. Interestingly, predictability (e.g., reliable information flow) was significantly associated with intentions to leave the homecare sector but not the current job in the organization. Peter et al. [74] did not find associations between predictability and turnover intention among hospital nurses in Switzerland. This might indicate that in contrast to hospital work, predictability may be more important in the homecare sector as a sector-specific issue, where care takes place at multiple locations, with less equipment and on-the-spot support available [75] and thereby be one possible approach for organizations to improve the homecare working environment. In contrary to the study of Rahnfeld et al. [24], social support by colleagues was associated with the intention to leave the current job in our sample, but not the homecare sector, even when controlled for job satisfaction. Although homecare workers mostly work alone at clients' homes, social support by colleagues also seems to play an important role in the homecare setting. However, both effects, i.e., predictability and social support by colleagues were small and may be due to the large sample size rather than clinical relevance.

Of the three work context factors explored in our study (i.e., overtime, role clarity, and role conflicts), we only found working overtime to be significantly associated to intention to leave the current job, which goes in line with the findings of previous studies [39, 70, 76, 77]. Our results and the result of Senek et al. [70] also imply that it depends, however, on how often and how long overtime has to be worked—the more often overtime is worked (compared to no overtime), the greater the association with the intention to leave. This result is an indication for organizations to look at the frequency of their employees' overtime, whereby a frequent occurrence of overtime should be avoided.

In alignment with the model of nurse turnover behavior [22], job satisfaction was significantly negatively associated to intention to leave and mediated the direct effect of several work environment and work context factors on respondents' intention to leave. This association has already been confirmed in previous homecare studies [9, 24, 27, 30]. Senek et al. [70] reported in their recent study that, of those homecare workers who intended to leave, 89% were dissatisfied with their job. As it was not our aim, we did not test whether the relationships between the independent variables and the mediator job satisfaction was significant, which has, however, been found in previous studies, for staffing [78], teamwork [79], leadership, and role conflicts [22]. These findings suggest that staffing, teamwork, or role conflicts have an indirect effect through job satisfaction on leaving intentions.

### 4.1. Strengths and Limitations

This analysis is based on a national sample of Swiss homecare agencies and their homecare workers. The large sample size, the high response rate, the use of validated scales, and the sensitivity analyses to check the robustness of the results strengthen the validity of the findings. Nevertheless, the generalizability of the findings is limited as homecare settings across the world are very different. In addition, the data were collected during the COVID-19 pandemic, which could have influenced employees' perceptions of their work environment and work context as well as intention to leave. The correlations with the intention to leave could differ according to the educational background of the homecare workers, as RNs have more responsibility and a larger scope of practice than LPNs, who are not allowed to conduct needs' assessments, or HCAs, who only provide basic care. However, this was not the focus of our study and we only controlled for this variable. Also, because of the cross-sectional data, causal relationships cannot be obtained. Since the data are based on self-report measures, neither response bias nor common method bias can be excluded. In addition, the internal consistency of the variables' social support from colleagues and role conflicts were low (*α* < 0.70), possibly because only two items each represented one scale. Also, unconsidered factors may have influenced our results, although the explained variance was high. Furthermore, by building a complete case dataset for the analysis, we could have increased the Type 2 error, which results in a loss of statistical power and affect the validity of the analysis [80].

## 5. Conclusions

Given the projected shortage of homecare workers, a major focus needs to be on how to keep homecare workers in their homecare profession, by both political decision-makers and homecare leadership. Based on the findings of this study, the quality of leadership appears to be one of the most important work environment factors related to intention to leave. Therefore, we recommend to examine if the leadership competencies or styles meet homecare workers' expectations. Leaders should consider practicing the transformative leadership style, which emphasizes development, improvement, and empowerment [72] and has shown to reduce intention to leave among employees significantly [71]. Based on our results, we also recommend that homecare agencies investigate the extent to which homecare workers are affected by overtime, and how predictability and social support from colleagues are experienced within their organization. Improvement efforts in these areas could help not only to reduce intention to leave but also to improve job satisfaction. Future studies could focus on deepening our understanding on how to sustain a positive work environment and how to empower nurse leaders in creating and sustaining a positive work environment [81].

## Figures and Tables

**Table 1:** Instruments and measurements of the independent variables and the mediator variable.

Variable	Instrument (reference)	Number of items/scale calculation	Items	Answer options	Cronbach's alpha^∗^/meaning of score
*Work environment factors*					
Leadership	PES-NWI [23]	5/mean over all items	- Supportive unit supervisor - Mistakes are used as learning opportunities - Competent leader - Praise and recognition for good work - Backup in decision-making	4-point Likert scale: 1 = *strongly disagree* to 4 = *strongly agree*	*α* = 0.84 [23], in homecare: *α* = 0.88 [53] Scores indicate better leadership quality [23]
Staffing	PES-NWI [23]	3/mean over all items	- Enough time to discuss client problems - Enough qualified personnel - Enough staff to get work done	4-point Likert scale: 1 = *strongly disagree* to 4 = *strongly agree*	*α* = 0.74 [23], in homecare: *α* = 0.83 [53] Scores indicate better staffing levels [23]
Teamwork	SAQ [47, 48]	6/mean over all items	- Input is well received - It is difficult to speak up about perceived problems - Disagreements are resolved appropriately - Getting support needed to care for clients - Asking questions is easy - Professionals work as a well-coordinated team	5-point Likert scale: 1 = *strongly disagree* to 5 = *strongly agree* with answer option “I do not know”	*α* = 0.65 [48], in homecare: *α* = 0.83 [53] Higher scores indicate better teamwork
Safety climate	SAQ [47, 48]	7/mean over all items	- As a client, I would feel safe - Errors are handled in an appropriate manner - Channels to direct questions about client safety - Appropriate feedback about performance - Difficulties to discuss errors in team - Encouraged to report client safety concerns - Culture of learning from errors	5-point Likert scale: 1 = *strongly disagree* to 5 = *strongly agree* with answer option “I do not know”	*α* = 0.75 [48] Higher scores indicate better safety climate
Predictability	COPSOQ III [49, 50]	2/mean over all items	- The extent to which good information is provided in advance - The extent to which all the information needed to do the work well is obtained	5-point Likert scale: 0 = *to a very small extent* to 100 = *to a very large extent*	*α* = 0.73 [49], in homecare: *α* = 0.80 [53] Higher scores indicate higher predictability
Social support from colleagues	COPSOQ III [49, 50]	2/mean over all items	- Frequency of getting help and support - Frequency of conversations about how well work was carried out	5-point Likert scale: 0 = *never/hardly* to 100 = *always* with answer option: “do not have colleagues”	*α* = 0.87 [49] Higher scores indicate higher social support from colleagues

*Work context factors*					
Role clarity	COPSOQ III [49, 50]	2/mean over all items	- To what extent work has clear objectives - To what extent area of responsibility is known	5-point Likert scale: 0 = *to a very small extent* to 100 = *to a very large extent*	*α* = 0.82 [49] Higher scores indicate better role clarity
Role conflicts	COPSOQ III [49, 50]	2/mean over all items	- To what extent contradictory demands are imposed - To what extent things have to be done that should have been done differently	5-point Likert scale: 0 = *to a very small extent* to 100 = *to a very large extent*	*α* = 0.73 [49] Higher scores indicate higher levels of role conflicts
Overtime	Adapted from RN4CAST study [51]	Single item	- How often more than 30 min of overtime work was required	5-point Likert scale: 0 = *never*, 1 = *less frequently*, 2 = *every 5–7 working days*, 3 = *every 2–4 working days*, 4 = *almost every shift*	N/A A higher score indicates that overtime is worked more often

*Mediator*					
Job satisfaction	COPSOQ II [50, 52]	4/mean over all items	- Overall job satisfaction - Satisfaction with job prospects - Satisfaction with physical working conditions - Satisfaction about how abilities are used	4-point Likert scale: 0 = *very dissatisfied* to 100 = *very satisfied*	*α* = 0.82 [52] Higher scores indicate higher job satisfaction

*Note*: COPSOQ III = Third Version of the Copenhagen Psychosocial Questionnaire, RN4CAST = Nurse Forecasting: Human Resources Planning in Nursing.

Abbreviations: N/A = not applicable, PES-NWI = Practice Environment Scale of the Nursing Work Index, SAQ = Safety Attitudes Questionnaire.

^∗^unless otherwise indicated *α* of original scale.

**Table 2:** Respondent characteristics of the complete case dataset used for the regression analysis (*n* = 1898).

**Variable**	**Complete cases (*n* = 1898)**			
	***n* (%)**	***M* (SD)**	**Range**	**Missing *n* (%)**

Employee characteristics	1898			

Gender				4 (0.2)^‡^
Female	1783 (94.1)			
Male	109 (5.8)			
Nonbinary	2 (0.1)			
Age (in years)		46.0 (11.9)	19–76	0 (0)
Educational background				0 (0)
Registered nurse (at least 3-4 years of tertiary level education)	856 (45.1)			
Licensed practical nurse (3-year vocational training)	602 (31.7)			
Homecare aide (certified course)	440 (23.2)			
Employment percentages (%)		63.4 (21.6)	5–100	0 (0)
≤ 40	397 (20.9)			
> 40 to ≤ 60	582 (30.7)			
> 60 to ≤ 80	645 (34.0)			
> 80	274 (14.4)			
Professional experience (in years)		16.8 (10.7)	0–47	98 (5.2)^‡^
≤ 2 years	82 (4.3)			
> 2 years ≤ 5 years	195 (10.3)			
> 5 years ≤ 10 years	385 (20.3)			
> 10 years ≤ 20 years	547 (28.8)			
> 20	591 (31.1)			
Experience in the current agency (in years)		6.9 (7.0)	0–43	75 (4.0)^‡^
≤ 2	600 (31.6)			
> 2 to ≤ 5	422 (22.3)			
> 5 to ≤ 10	405 (21.3)			
> 10 to ≤ 20	295 (15.5)			
> 20	101 (5.3)			
Compensation				0 (0)
Hourly wage	370 (19.5)			
Annual salary	1528 (80.5)			

Agency characteristics	85			

Profit status				0 (0)
Public nonprofit homecare agency	14 (16.5)			
Private nonprofit homecare agency	47 (55.3)			
Private for-profit homecare agency	24 (28.2)			
Size of the homecare agency:				
Number of full-time equivalents		86.5 (76.1)	4.7–318	0 (0)
Number of clients per year		1057 (928.6)	7–3478	0 (0)
Catchment area of homecare agency				0 (0)
Rural	37 (43.5)			
Suburban	32 (37.7)			
Urban	16 (18.8)			

*Note: M* = mean and *n* = number.

Abbreviation: SD = standard deviation.

^‡^The complete case dataset is based on the variables used for the statistical models. The variables' gender, professional experience, or experience in the current agency was not included in the models.

**Table 3:** Characteristics of the dependent, independent, and mediator variables of the complete cases dataset (*n* = 1898).

Variable (range of response options)	Complete cases (*n* = 1898)	
	*n* (%)	*M* (SD)
*Dependent*		
Intention to leave the current job (0–4)		
Intention to leave the current job (dichotomized)		
Fully disagreed (sum score = 0)	789 (41.6)	
Agreed at least to a slight intention (sum score ≥ 1)	1109 (58.4)	
Intention to leave the homecare sector (0–4)^‡^		
Strongly disagreed	942 (49.6)	
Slightly disagreed	406 (21.4)	
Neutral	323 (17.0)	
Slightly agreed	144 (7.6)	
Strongly agreed	83 (4.4)	
Intention to leave the homecare sector (dichotomized)		
Disagreed	1671 (88.0)	
Agreed	227 (12.0)	

*Independent*		
Leadership (1–4)		3.3 (0.6)
Staffing (1–4)		2.9 (0.7)
Teamwork (1–5)		4.3 (0.7)
Safety climate (1–5)		4.1 (0.7)
Predictability (0–100)		65.0 (18.9)
Social support colleagues (0–100)		62.7 (20.5)
Role clarity (0–100)		76.2 (18.0)
Role conflicts (0–100)		30.5 (21.5)
Overtime (1–5)^‡^		
Never	90 (4.7)	
Less frequently	578 (30.5)	
Every 5–7 working days	416 (22.0)	
Every 2–4 working days	517 (27.2)	
Almost every shift	297 (15.6)	

*Mediator*		
Job satisfaction (0–100)		67.5 (15.0)

*Note*: *M* = mean and *n* = number.

Abbreviations: CI = confidence interval, N/A = not applicable, and SD = standard deviation.

^‡^Single-item variable.

**Table 4:** Structural factors associated with respondents' intention to leave the current job (*n* =* *1898).

Variable or effect size	Univariate multilevel models^†^		GLMM adjusted^‡^		GLMM adjusted^‡^, including job satisfaction	
	OR	95% CI	OR	95% CI	OR	95% CI
Work environment						
Leadership	0.19^∗∗∗^	[0.15, 0.23]	0.42^∗∗∗^	[0.32, 0.55]	0.48^∗∗∗^	[0.36, 0.65]
Staffing	0.29^∗∗∗^	[0.25, 0.35]	0.75^∗^	[0.60, 0.94]	0.93	[0.73, 1.18]
Teamwork	0.33^∗∗∗^	[0.28, 0.40]	0.84	[0.64, 1.09]	0.90	[0.68, 1.19]
Safety climate	0.31^∗∗∗^	[0.26, 0.37]	0.85	[0.65, 1.11]	0.91	[0.69, 1.21]
Predictability	0.96^∗∗∗^	[0.95, 0.96]	0.99^∗∗∗^	[0.98, 0.99]	0.99	[0.98, 1.00]
Social support colleagues	0.98^∗∗∗^	[0.97, 0.98]	0.99^∗∗^	[0.98, 1.00]	0.99^∗^	[0.99, 1.00]
Work context						
Role clarity	0.96^∗∗∗^	[0.96, 0.97]	0.99	[0.98, 1.00]	0.99	[0.99, 1.00]
Role conflicts	1.02^∗∗∗^	[1.02, 1.03]	1.01^∗^	[1.00, 1.01]	1.00	[1.00, 1.01]
Overtime (reference: never)						
Almost every shift	3.20^∗∗∗^	[1.91, 5.35]	2.20^∗^	[1.18, 4.11]	2.24^∗^	[1.15, 4.38]
Every 2–4 working days	2.48^∗∗∗^	[1.54, 4.02]	1.86^∗^	[1.05, 3.31]	2.05^∗^	[1.11, 3.82]
Every 5–7 working days	1.74^∗^	[1.07, 2.83]	1.47	[0.83, 2.61]	1.60	[0.86, 2.96]
Less frequently	1.57^§^	[0.98, 2.51]	1.54	[0.89, 2.66]	1.68	[0.93, 3.04]
Mediator						
Job satisfaction					0.93^∗∗∗^	[0.91, 0.94]
Effect size						
AIC				2116		1913
Marginal *R*^2^				0.35		0.50
Conditional *R*^2^				0.37		0.52

*Note*: α-level for significance: ^∗^*p* < 0.05, ^∗∗^*p* < 0.01, and ^∗∗∗^*p* < 0.001.

Abbreviations: AIC = Akaike Information Criterion, CI = confidence interval, GLMM = generalized linear mixed model, and OR = odds ratio.

^†^The univariate multilevel models were used for initial variable selection (screening criterion *p* < 0.20).

^§^These variables met the screening criterion *p* < 0.20, although *p* ≥ 0.05.

^‡^The adjusted GLMM was controlled for individual factors: educational background, age, employment percentages, compensation, and agency factors such as agency size (i.e., number of full-time equivalents and number of clients per year), catchment area, and profit status.

**Table 5:** Structural factors associated with respondents' intention to leave the homecare sector (*n* = 1898).

Variable/effect size	Univariate multilevel models^†^		GLMM adjusted^‡^		GLMM adjusted^‡^, including job satisfaction	
	OR	95% CI	OR	95% CI	OR	95% CI
Work environment						
Leadership	0.35^∗∗∗^	[0.28, 0.44]	0.59^∗∗∗^	[0.44, 0.80]	0.72^∗^	[0.53, 0.99]
Staffing	0.38^∗∗∗^	[0.31, 0.47]	0.70^∗^	[0.53, 0.92]	0.83	[0.61, 1.11]
Teamwork	0.49^∗∗∗^	[0.40, 0.60]	0.75	[0.54, 1.04]	0.86	[0.61, 1.21]
Safety climate	0.51^∗∗∗^	[0.42, 0.63]	1.26	[0.89, 1.79]	1.30	[0.90, 1.88]
Predictability	0.97^∗∗∗^	[0.96, 0.97]	0.98^∗∗∗^	[0.97, 0.99]	0.99^∗^	[0.98, 1.00]
Social support colleagues	0.99^∗∗∗^	[0.98, 0.99]	1.00	[0.99, 1,01]	1.00	[0.99, 1.01]
Work context						
Role clarity	0.98^∗∗∗^	[0.97, 0.99]	1.00	[0.99, 1,01]	1.00	[0.99, 1.02]
Role conflicts	1.02^∗∗∗^	[1.02, 1.03]	1.01	[1.00, 1,01]	1.00	[1.00, 1.01]
Overtime (reference: never)						
Almost every shift	2.12^§^	[0.95, 4.76]	1.22	[0.49, 3.04]	1.33	[0.50, 3.52]
Every 2–4 working days	1.94^§^	[0.89, 4.24]	1.52	[0.64, 3.65]	1.90	[0.76, 4.82]
Every 5–7 working days	1.43	[0.65, 3.18]	1.29	[0.57, 3.36]	1.76	[0.69, 4.50]
Less frequently	0.83	[0.37, 1.84]	0.85	[0.35, 2.05]	1.03	[0.40, 2.60]
Mediator						
Job satisfaction					0.95^∗∗∗^	[0.94, 0.96]
Effect size						
AIC				1254		1185
Marginal *R*^2^				0.24		0.31
Conditional *R*^2^				0.26		0.33

*Note:* α-level for significance: ^∗^*p* < 0.05, ^∗∗^*p* < 0.01, and ^∗∗∗^*p* < 0.001.

Abbreviations: AIC = Akaike Information Criterion, CI = confidence interval, GLMM = generalized linear mixed model, and OR = odds ratio.

^†^The univariate multilevel models were used for initial variable selection (screening criterion *p* < 0.20).

^§^These variables met the screening criterion *p* < 0.20, although *p* ≥ 0.05.

^‡^The adjusted GLMM was controlled for individual factors: educational background, age, employment percentages, compensation, and agency factors such as agency size (i.e., number of full-time equivalents and number of clients per year), catchment area, and profit status.

## Data Availability

The data that support the findings of this study are available on request from the corresponding author. The data are not publicly available due to privacy or ethical restrictions.

## Supporting Information

Supporting Information 1 Appendix A contains the respondent characteristics of the total subsample (*n* = 2240) and complete cases' dataset (*n* = 1898) in comparison.

Supporting Information 2 Appendix B contains the sensitivity analysis for respondents' intention to leave the job and the homecare sector.
