# Supplementary material for: Work Environment and Work Context Factors Associated With Homecare Workers' Intention to Leave: An Analysis of a National, Multicenter Cross-Sectional Study
Source: J Nurs Manag. 2025 Jul 9;2025:1554741. doi: 10.1155/jonm/1554741 (PMC12267978; doi:10.1155/jonm/1554741)
Supplement: Supporting Information 1 — Appendix A contains the respondent characteristics of the total subsample (n = 2240) and complete cases' dataset (n = 1898) in comparison. [file 1554741.f1.docx]

#

# Appendix A. Comparison of the *total sub-sample (n = 2,240) and complete cases dataset (n = 1,898)*

**Table 1.** *Respondent characteristics of total sub-sample fulfilling inclusion criteria (n = 2,240) and the complete cases dataset used for the regression analysis (n = 1,898)*

|  |  | **Sub-sample total (n = 2,240)** | | | |  | **Complete cases (n = 1,898)** | | | |
| --- | --- | --- | --- | --- | --- | --- | --- | --- | --- | --- |
| **Variable** |  | ***n* (%)** | ***M* (*SD*)** | **Range** | **Missing’s  *n* (%)** |  | ***n* (%)** | ***M* (*SD*)** | **Range** | **Missing’s  *n* (%)** |
| **Employee characteristics** |  | **2,240** |  |  |  |  | **1,898** |  |  |  |
| Gender |  |  |  |  | 8 (0.4) |  |  |  |  | 4 (0.2) ^‡^ |
| Female |  | 2,101 (94.1) |  |  |  |  | 1,783 (94.1) |  |  |  |
| Male |  | 127 (5.7) |  |  |  |  | 109 (5.8) |  |  |  |
| Non-binary |  | 4 (0.2) |  |  |  |  | 2 (0.1) |  |  |  |
| Age (in years) |  |  | 46.3 (12.0) | 19-77 | 66 (2.9) |  |  | 46.0 (11.9) | 19-76 | 0 (0) |
| Educational background |  |  |  |  | 0 (0) |  |  |  |  | 0 (0) |
| Registered nurse (at least 3-4 years of tertiary level education) |  | 961 (42.9) |  |  |  |  | 856 (45.1) |  |  |  |
| Licensed practical nurse (3-year vocational training) |  | 713 (31.8) |  |  |  |  | 602 (31.7) |  |  |  |
| Homecare aide (certified course) |  | 566 (25.3) |  |  |  |  | 440 (23.2) |  |  |  |
| Employment percentages (%) |  |  | 63.4 (21.8) | 5-100 | 53 (2.4) |  |  | 63.4 (21.6) | 5-100 | 0 (0) |
| ≤ 40 |  | 456 (20.4) |  |  |  |  | 397 (20.9) |  |  |  |
| > 40 to ≤ 60 |  | 671 (30.0) |  |  |  |  | 582 (30.7) |  |  |  |
| > 60 to ≤ 80 |  | 740 (33.0) |  |  |  |  | 645 (34.0) |  |  |  |
| > 80 |  | 320 (14.3) |  |  |  |  | 274 (14.4) |  |  |  |
| Professional experience (in years) |  |  | 16.7 (10.8) | 0-53 | 150 (6.7) |  |  | 16.8 (10.7) | 0-47 | 98 (5.2) ^‡^ |
| ≤ 2 years |  | 103 (4.9) |  |  |  |  | 82 (4.3) |  |  |  |
| > 2 years ≤ 5 years |  | 232 (11.1) |  |  |  |  | 195 (10.3) |  |  |  |
| > 5 years ≤ 10 years |  | 443 (21.2) |  |  |  |  | 385 (20.3) |  |  |  |
| > 10 years ≤ 20 years |  | 635 (30.4) |  |  |  |  | 547 (28.8) |  |  |  |
| > 20 |  | 677 (32.4) |  |  |  |  | 591 (31.1) |  |  |  |
| Experience in current agency (in years) |  |  | 6.9 (7.0) | 0-43 | 122 (5.4) |  |  | 6.9 (7.0) | 0-43 | 75 (4.0) ^‡^ |
| ≤ 2 |  | 698 (33.0) |  |  |  |  | 600 (31.6) |  |  |  |
| > 2 to ≤ 5 |  | 491 (23.2) |  |  |  |  | 422 (22.3) |  |  |  |
| > 5 to ≤ 10 |  | 475 (22.4) |  |  |  |  | 405 (21.3) |  |  |  |
| > 10 to ≤ 20 |  | 338 (16.0) |  |  |  |  | 295 (15.5) |  |  |  |
| > 20 |  | 116 (5.5) |  |  |  |  | 101 (5.3) |  |  |  |
| Compensation |  |  |  |  | 17 (0.8) |  |  |  |  | 0 (0) |
| Hourly wage |  | 482 (21.7) |  |  |  |  | 370 (19.5) |  |  |  |
| Annual salary |  | 1,741 (78.3) |  |  |  |  | 1,528 (80.5) |  |  |  |
| **Agency characteristics** |  | **88** |  |  |  |  | **85** |  |  |  |
| Profit status |  |  |  |  | 0 (0) |  |  |  |  | 0 (0) |
| Public nonprofit homecare agency |  | 15 (17.1) |  |  |  |  | 14 (16.5) |  |  |  |
| Private nonprofit homecare agency |  | 47 (53.4) |  |  |  |  | 47 (55.3) |  |  |  |
| Private for-profit homecare agency |  | 26 (29.5) |  |  |  |  | 24 (28.2) |  |  |  |
| Size of the homecare agency: |  |  |  |  |  |  |  |  |  |  |
| Number of full-time equivalents |  |  | 84.9 (76.1) | 4.7-318 | 0 (0) |  |  | 86.5 (76.1) | 4.7-318 | 0 (0) |
| Number of clients per year |  |  | 1,046.8 (927.3) | 7-3,478 | 58 (2.6) |  |  | 1,057 (928.6) | 7-3,478 | 0 (0) |
| Catchment area of homecare agency |  |  |  |  | 0 (0) |  |  |  |  | 0 (0) |
| Rural |  | 39 (44.3) |  |  |  |  | 37 (43.5) |  |  |  |
| Suburban |  | 32 (36.4) |  |  |  |  | 32 (37.7) |  |  |  |
| Urban |  | 17 (19.3) |  |  |  |  | 16 (18.8) |  |  |  |

*Note.* *M* = mean, n = number, *SD* = standard deviation

^‡^ The complete case dataset is based on the variables used for the statistical models. The variables gender, professional experience, or experience in current agency were not included in the models.

**Table 2.** *Characteristics of the dependent, independent and mediator variables, total sub-sample (n = 2,240) and complete cases dataset (n = 1,898)*

|  |  | **Sample total (n = 2,240)** | | |  | **Complete cases (n = 1,898)** | |
| --- | --- | --- | --- | --- | --- | --- | --- |
| **Variable (range of response options)** | ***n* (%)** | ***M* (*SD*)** | **Cronbach’s α [95% CI]** | **Missing’s *n* (%)** |  | ***n* (%)** | ***M* (*SD*)** |
| **Dependent** |  |  |  |  |  |  |  |
| Intention to leave the current job (0-4) |  | 0.85 (1.0), | .90 [.89, .91] | 54 (2.4) |  |  |  |
| Intention to leave the current job (dichotomized) |  |  |  |  |  |  |  |
| Fully disagreed (sum score = 0)  Agreed at least to a slight intention (sum score ≥ 1) | 896 (41.0)  1290 (59.0) |  |  |  |  | 789 (41.6)  1109 (58.4) |  |
| Intention to leave the homecare sector (0-4) ^‡^ |  |  | N/A | 44 (2.0) |  |  |  |
| Strongly disagreed  Slightly disagreed  Neutral  Slightly agreed  Strongly agreed | 1078 (49.1)  468 (21.3)  373 (17.0)  173 (7.9)  104 (4.7) |  |  |  |  | 942 (49.6)  406 (21.4)  323 (17.0)  144 (7.6)  83 (4.4) |  |
| Intention to leave the homecare sector (dichotomized) |  |  |  |  |  |  |  |
| Disagreed  Agreed | 1919 (87.4)  277 (12.6) |  |  |  |  | 1671 (88.0)  227 (12.0) |  |
| **Independent** |  |  |  |  |  |  |  |
| Leadership (1-4) |  | 3.3 (0.6) | .88 [.86, .88] | 38 (1.7) |  |  | 3.3 (0.6) |
| Staffing (1-4) |  | 2.9 (0.7) | .72 [.70, .75] | 16 (0.7) |  |  | 2.9 (0.7) |
| Teamwork (1-5) |  | 4.3 (0.7) | .85 [.83, .86] | 14 (0.6)^§^ |  |  | 4.3 (0.7) |
| Safety climate (1-5) |  | 4.1 (0.7) | .86 [.85, .87] | 8 (0.4)^§^ |  |  | 4.1 (0.7) |
| Predictability (0-100) |  | 65.1 (18.9) | .74 [.71, .76] | 14 (0.6) |  |  | 65.0 (18.9) |
| Social support colleagues (0-100) |  | 62.3 (20.6) | .61 [.57, .64] | 20 (0.9)^§^ |  |  | 62.7 (20.5) |
| Role clarity (0-100) |  | 76.2 (18.1) | .72 [.69, .75] | 11 (0.5) |  |  | 76.2 (18.0) |
| Role conflicts (0-100) |  | 30.6 (21.5) | .69 [.66, .72] | 22 (1.0) |  |  | 30.5 (21.5) |
| Overtime (1-5) ^‡^ |  |  | N/A | 31 (1.4) |  |  |  |
| Never  Less frequently  Every 5-7 working days  Every 2-4 working days  Almost every shift | 115 (5.2)  714 (32.3)  478 (21.6)  568 (25.7)  334 (15.1) |  |  |  |  | 90 (4.7)  578 (30.5)  416 (22.0)  517 (27.2)  297 (15.6) |  |
| **Mediator** |  |  |  |  |  |  |  |
| Job satisfaction (0-100) |  | 67.5 (14.9) | .74 [.72, .77] | 55 (2.5) |  |  | 67.5 (15.0) |

*Note*. CI = confidence interval, *M* = mean, n = number, N/A = not applicable*, SD* = standard deviation.

^‡^ Single item variable.

^§^ Missing values include the answer options “I do not know” or “I do not have colleagues”.
