# Supplementary material for: Work Environment and Work Context Factors Associated With Homecare Workers' Intention to Leave: An Analysis of a National, Multicenter Cross-Sectional Study
Source: J Nurs Manag. 2025 Jul 9;2025:1554741. doi: 10.1155/jonm/1554741 (PMC12267978; doi:10.1155/jonm/1554741)
Supplement: Supporting Information 2 — Appendix B contains the sensitivity analysis for respondents' intention to leave the job and the homecare sector. [file 1554741.f2.docx]

# Appendix B. Sensitivity analysis for respondents’ intention to leave the job and the homecare sector

We conducted a sensitivity analysis for the outcome variable “intention to leave the job” and “intention to leave the homecare sector” by running linear multilevel regression models. In the first model we only included the control variables, in the second model also the mediator variable “job satisfaction.

## Linear multilevel regression with respondents’ intention to leave the job

To run the linear regression, we first calculated a mean score over the 3 items of the “intention to leave the job” scale. These values were used as dependent variable in the linear regression.

Mean value for the “intention to leave the job” scale was 0.85 (standard deviation 1.0) and the median was 0.33 with values ranging from 0-4.

**Table 1.** *Results of the linear multilevel regression with “intention to leave the job” (n = 1,898)*

|  |  | **LMM adjusted^‡^** | |  | **LMM adjusted ^‡^, including  job satisfaction** | |
| --- | --- | --- | --- | --- | --- | --- |
| **Variable or effect size** |  | **Beta coefficient** | **95% CI** |  | **Beta coefficient** | **95% CI** |
| **Work environment** |  |  |  |  |  |  |
| Leadership |  | -0.36*** | [-0.45, -0.27] |  | -0.25*** | [-0.33, -0.16] |
| Staffing |  | -0.12** | [-0.20, -0.04] |  | -0.03 | [-0.10, 0.05] |
| Teamwork |  | -0.09 | [-0.19, 0.00] |  | -0.03 | [-0.12, 0.06] |
| Safety climate |  | -0.05 | [-0.15, 0.04] |  | -0.03 | [-0.12, 0.06] |
| Predictability |  | -0.01*** | [-0.01, -0.00] |  | -0.00* | [-0.01, -0.00] |
| Social support colleagues |  | -0.00** | [-0.01, -0.00] |  | -0.00 | [-0.00, 0.00] |
| **Work context** |  |  |  |  |  |  |
| Role clarity |  | -0.00 | [-0.00, 0.00] |  | 0.00 | [-0.00, 0.00] |
| Role conflicts |  | 0.00 | [0.00, 0.00] |  | 0.00 | [-0.00, 0.00] |
| Overtime  [Reference: Never] |  |  |  |  |  |  |
| - Less frequently |  | 0.04 | [-0.16, 0.24] |  | 0.07 | [-0.11, 0.26] |
| - Every 5-7 working days |  | 0.00 | [-0.21, 0.21] |  | 0.04 | [-0.15, 0.23] |
| - Every 2-4 working days |  | 0.10 | [-0.11, 0.31] |  | 0.12 | [-0.07, 0.31] |
| - Almost every shift |  | 0.18 | [-0.04, 0.40] |  | 0.16 | [-0.05, 0.37] |
| **Mediator variable** |  |  |  |  |  |  |
| Job satisfaction |  |  |  |  | -0.03*** | [-0.03, -0.03] |
| **Effect size** |  |  |  |  |  |  |
| AIC |  |  | 5,113 |  |  | 4,828 |
| Marginal *R^2^* Conditional *R^2^* |  |  | 0.27  0.30 |  |  | 0.38  0.40 |

*Note*. AIC = Akaike Information Criterion, CI = Confidence Interval, LMM = Linear Multilevel Model.

^‡^The LMM adjusted was controlled for: educational background, age, employment percentages, compensation, agency size (i.e., number of full-time equivalents and number of clients per year), catchment area and profit status.

α-level for significance: **p* < .05. ***p* < .01. ****p* < .001

## Linear multilevel regression with respondents’ intention to leave the homecare sector

To run the linear regression, we used the single item variable “intention to leave homecare sector” as dependent variable in the linear regression. Values ranged from 0-4 (see Table 3 in the article for more details).

**Table 2.** *Results of the linear multilevel regression with “intention to leave the homecare sector” (n = 1,898)*

|  |  | **LMM adjusted^‡^** | |  | **LMM adjusted ^‡^, including  job satisfaction** | |
| --- | --- | --- | --- | --- | --- | --- |
| **Variable or effect size** |  | **Beta coefficient** | **95% CI** |  | **Beta coefficient** | **95% CI** |
| **Work environment** |  |  |  |  |  |  |
| Leadership |  | -0.27*** | [-0.38, -0.16] |  | -0.16** | [-0.27, -0.06] |
| Staffing |  | -0.17*** | [-0.27, -0.07] |  | -0.08 | [-0.18, 0.01] |
| Teamwork |  | -0.16** | [-0.27, -0.04] |  | -0.10 | [-0.21, 0.01] |
| Safety climate |  | 0.06 | [-0.06, 0.18] |  | 0.08 | [-0.03, 0.20] |
| Predictability |  | -0.01*** | [-0.01, -0.00] |  | -0.00 | [-0.01, 0.00] |
| Social support colleagues |  | -0.00 | [-0.01, 0.00] |  | -0.00 | [-0.00, 0.00] |
| **Work context** |  |  |  |  |  |  |
| Role clarity |  | -0.00 | [-0.01, 0.00] |  | -0.00 | [-0.00, 0.00] |
| Role conflicts |  | 0.00 | [-0.00, 0.00] |  | 0.00 | [-0.00, 0.00] |
| Overtime  [Reference: Never] |  |  |  |  |  |  |
| - Less frequently |  | 0.08 | [-0.16, 0.32] |  | 0.11 | [-0.12, 0.34] |
| - Every 5-7 working days |  | 0.15 | [-0.10, 0.41] |  | 0.19 | [-0.05, 0.43] |
| - Every 2-4 working days |  | 0.23 | [-0.02, 0.48] |  | 0.25* | [0.02, 0.49] |
| - Almost every shift |  | 0.25 | [-0.02, 0.52] |  | 0.23 | [-0.02, 0.49] |
| **Mediator variable** |  |  |  |  |  |  |
| Job satisfaction |  |  |  |  | -0.03*** | [-0.03, -0.02] |
| **Effect size** |  |  |  |  |  |  |
| AIC |  |  | 5,803 |  |  | 5,630 |
| Marginal *R^2^* Conditional *R^2^* |  |  | 0.18  0.19 |  |  | 0.25  0.27 |

*Note*. AIC = Akaike Information Criterion, CI = Confidence Interval, LMM = Linear Multilevel Model.

^‡^The LMM adjusted was controlled for: educational background, age, employment percentages, compensation, agency size (i.e., number of full-time equivalents and number of clients per year), catchment area and profit status.

α-level for significance: **p* < .05. ***p* < .01. ****p* < .001
